# A Perspective on Multiscale Electrodiffusion and Model Selection for Ion Transport in Energy-Storage Nanomaterials

**DOI:** 10.3390/nano16171124

**Published:** 2026-09-07

**Authors:** Hamid Mofidi, Wuyue Yang, Mingji Zhang

**Affiliations:** 1 Beijing Institute of Mathematical Sciences and Applications (BIMSA), Beijing 101408, China; yangwuyue@bimsa.cn; 2 Yau Mathematical Sciences Center (YMSC), Tsinghua University, Beijing 100084, China; 3 Department of Mathematics, New Mexico Institute of Mining and Technology, Socorro, NM 87801, USA; mingji.zhang@nmt.edu

**Keywords:** ion transport, energy storage, nanomaterials, model selection, Poisson–Nernst–Planck equations, Doyle–Fuller–Newman model, finite ion size, space charge, porous electrodes, machine learning

## Abstract

Ion transport in energy storage nanomaterials is described with models that resolve different scales and different physical effects. Device models such as the Doyle–Fuller–Newman framework are well suited to cell voltage, concentration polarization, and electrode utilization, but they usually average local charge separation into effective transport and interface laws. Charge-resolved Poisson–Nernst–Planck, modified Poisson–Nernst–Planck, and Poisson–Fermi descriptions can instead resolve electric potential, ion partitioning, finite ion size, and space charge inside narrow pores and interphases. Molecular simulation gives solvation, adsorption, and interfacial structure, while pore-network and homogenized models connect local transport to electrode geometry. This Perspective compares these model families, states their limits, and explains how quantities should be passed from molecular and pore scales to electrode and device models. Representative examples are discussed for liquid-electrolyte batteries, all-solid-state batteries, and nanoporous supercapacitors. Two concise calculations are retained as model audits. The first tests whether a first-order finite-size approximation remains reliable when its leading correction is small by cancellation. The second tests when local electroneutrality becomes inaccurate in a charged pore and how steric crowding changes screening. These calculations are not new device predictions. The main contribution is a practical procedure that links model choice, cross-scale handoff, and validation with measurable quantities such as voltage, concentration profiles, impedance, interfacial resistance, capacitance, and charging time.

## 1. Introduction

Electrochemical energy storage depends on the motion of charged species through liquids, solids, pores, and interfaces. In lithium-ion and sodium-ion batteries, solid-state batteries, and supercapacitors, measured quantities such as voltage, capacity, rate capability, impedance, and charging time arise from processes that span several length and time scales. These processes include solvation and desolvation, diffusion and migration, adsorption and insertion, phase change, interphase growth, and changes in pore access. Nanostructuring shortens some transport paths and increases active surface area, but it also makes local electric fields, surface charge, crowding, and interfacial structure more important [1,2,3,4].

The main modeling question is not which equation is most detailed. It is which variables must be resolved for the observable and operating regime under study. A Doyle–Fuller–Newman model can predict cell voltage and electrode utilization after local transport and interface processes have been represented by effective parameters. A charge-resolved continuum model can examine potential and concentration profiles inside a narrow pore or a space-charge region, but it does not by itself describe a full commercial cell. Molecular simulation can reveal solvation, adsorption, and ionic layering, but its limited size and time scales require an explicit handoff to coarser models. Pore-network and homogenized descriptions provide intermediate steps by converting local laws and microstructure into effective conductivity, diffusivity, capacitance, and reaction terms [5,6,7].

Published studies show why this division of labor is needed. Parameterized porous-electrode models have reproduced discharge and drive-cycle responses of commercial lithium-ion cells when the required thermodynamic, kinetic, and transport data are measured carefully [8,9]. Operando X-ray fluorescence has also measured solution-phase concentration profiles in a working porous electrode and provides a direct target for electrolyte-transport validation [10]. In all-solid-state batteries, charge-density variations across a cathode–electrolyte interface have been observed by in situ microscopy and compared with finite-element calculations, while Poisson–Fermi–Dirac models have connected defect chemistry, band bending, and interfacial barriers [11,12]. In supercapacitors, molecular simulations can reproduce capacitance in realistic nanoporous carbons, and combined simulation and experiment have connected pore structure, electrolyte composition, and charging dynamics [13,14,15].

This Perspective organizes these descriptions around model selection and cross-scale transfer. Poisson–Nernst–Planck equations receive particular attention because they make the relation among diffusion, migration, electric potential, and fixed charge explicit. Mathematical results developed for narrow charged systems, including ion channels and membranes, are used only where they clarify general issues such as boundary layers, finite ion size, current continuity, and sensitivity to charge distribution. Energy storage materials remain the main application throughout this article.

**Figure 1:**
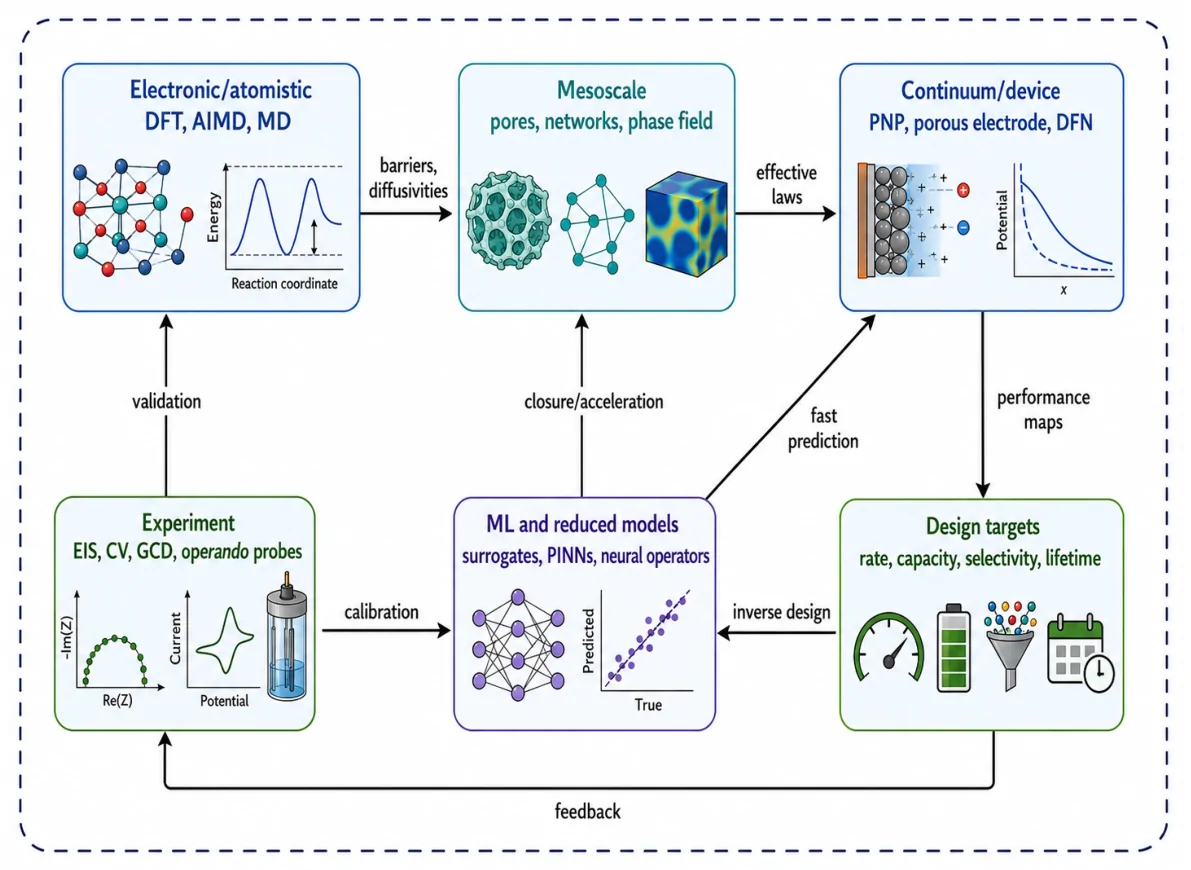
A multiscale loop for ion transport in energy storage nanomaterials. Electronic and atomistic calculations estimate local processes and parameters. Mesoscale descriptions encode pore geometry and microstructure. Continuum equations predict electrode and device response. Experiments constrain hidden variables and test model outputs. Machine-learning and reduced-order methods support simulation, parameter estimation, and design after suitable validation. Blue boxes denote electronic/atomistic and continuum/device descriptions, teal denotes mesoscale descriptions, green denotes experiment and design targets, and purple denotes machine-learning and reduced-model components. Arrows indicate the direction of information transfer. The abbreviations are DFT for density functional theory, AIMD for ab initio molecular dynamics, MD for molecular dynamics, PNP for Poisson–Nernst–Planck, DFN for Doyle–Fuller–Newman, EIS for electrochemical impedance spectroscopy, CV for cyclic voltammetry, GCD for galvanostatic charge–discharge, ML for machine learning, and PINNs for physics-informed neural networks.

The workflow is summarized in Figure 1. Smaller-scale calculations and experiments supply parameters and constitutive laws. Mesoscale descriptions represent geometry and connectivity. Electrode and device equations predict operating responses. Reduced and learning-based methods accelerate selected parts of this chain only after the physical variables and validation targets have been identified.

### Scope, Novelty, and Organization

This article is a Perspective. It does not report a new battery chemistry, a new experimental campaign, a full device simulation, or a trained machine-learning model. Its novelty is methodological. It brings together model choice, charge representation, cross-scale handoff, and observable-based validation in one procedure for nanostructured energy storage systems.

This article makes four main contributions. First, it directly compares porous-electrode and concentrated-solution theory, classical and modified Poisson–Nernst–Planck equations, Poisson–Fermi descriptions, molecular simulation, and pore upscaling. Second, it separates fixed charge, regulated charge, and dielectric-induced charge so that these effects are not counted twice. Third, it shows how local outputs such as ion partitioning, space-charge resistance, differential capacitance, and pore conductivity can enter an electrode or device model. Fourth, it states practical validation routes for liquid-electrolyte batteries, all-solid-state batteries, and supercapacitors. The two calculations in Section 5 serve as model-audit examples rather than new material predictions.

The literature was assembled through targeted searches and backward citation tracing rather than a database-counting systematic protocol. The main period considered is approximately 2000 through June 2026; studies from this period were considered together with earlier work on porous-electrode theory, electrodiffusion, and ion-selective transport. The scope includes transport equations, finite ion size, concentrated electrolytes, fixed and surface charge, solid electrolytes, interphases, homogenization, inverse problems, and learning-based reduced models. This procedure does not support bibliometric or exhaustive-coverage claims.

No new experimental data are presented. The discussion of validation is based on published measurements and simulations. Four questions guide the article: Which scale and observable are being modeled? Which physical effects are resolved or averaged? Which quantities are passed to the next scale? Which independent measurements can reject or support the chosen description?

Table 1 gives an initial summary of the model classes.

The remainder of this article is organized as follows. Section 2 states the main transport equations and regime indicators. Section 3 presents representative energy storage cases and model handoffs. Section 4 identifies useful mathematical ideas and the limits of the ion-channel analogy. Section 5 gives two concise model checks. Section 6 reviews recent uses of machine learning and gives a validation checklist. Section 7 connects model outputs with experiments. Section 8 summarizes the remaining challenges, and Section 9 concludes the article.

## 2. Mathematical Foundations of Ion Transport

This section fixes the notation and transport regimes used later in this article. It does not derive every transport equation; instead, it identifies the assumptions that change when nanoscale confinement, fixed charge, finite ion size, high concentration, or interfacial charge separation becomes important. The basic flux relation is introduced first, followed by Poisson coupling, dimensionless regime indicators, finite-size corrections, concentrated-electrolyte effects, and boundary-layer behavior.

### 2.1. Electrochemical Potential and the Nernst–Planck Flux

For an ionic species *i* with concentration $c_{i}$, valence $z_{i}$, diffusion coefficient $D_{i}$, and electrostatic potential ϕ, the classical Nernst–Planck flux can be written as

(1) $$J_{i}=-D_{i}\left(\nabla c_{i}+\frac{z_{i}e}{k_{B}T}c_{i}\nabla \phi \right),$$

where *e* is the elementary charge and $k_{B}T$ is the thermal energy. Under a diagonal Nernst–Einstein mobility approximation, the flux can be written in terms of the electrochemical-potential gradient,

(2) $$J_{i}=-\frac{D_{i}c_{i}}{k_{B}T}\nabla \mu_{i},\;\mu_{i}=\mu_{i}^{0}+k_{B}T\ln\;\left(\frac{c_{i}}{c^{\circ }}\right)+z_{i}e\phi +\mu_{i}^{\mathrm{ex}},$$

where $c^{\circ }$ is the standard-state concentration and $\mu_{i}^{\mathrm{ex}}$ encodes nonideal effects arising from finite ion volume, ion–ion correlations, solvation, or interactions with a host matrix. This formulation is useful because the same flux–potential coupling appears in dilute electrolytes, ion channels, membranes, and porous electrodes, while the constitutive form of $\mu_{i}^{\mathrm{ex}}$ changes with the physical regime. In concentrated environments, moreover, mass action cannot be interpreted in an ideal dilute sense unless the excess chemical potential is represented explicitly, a point emphasized repeatedly in work on crowded and interacting ionic solutions [16,17,18].

The diagonal form in Equation (2) assumes that the flux of species *i* responds directly to its own electrochemical-potential gradient. In a concentrated multicomponent electrolyte, the fluxes may also be coupled through the thermodynamic forces acting on the other species. In a selected reference frame, this coupling can be written in Onsager form as

(3) $$J_{i}-c_{i}v_{\mathrm{ref}}=-\sum_{j}L_{ij}\nabla \mu_{j},$$

where $v_{\mathrm{ref}}$ is the selected reference velocity and $L_{ij}$ are multicomponent mobility coefficients. The off-diagonal coefficients represent transport coupling between different species. Equivalent Stefan–Maxwell formulations express this coupling through relative velocities and interspecies friction coefficients. Thus, the excess chemical potential and the multicomponent mobility relation describe different aspects of concentrated-electrolyte transport.

Mass conservation gives

(4) $$\frac{\partial c_{i}}{\partial t}+\nabla \cdot J_{i}=R_{i},$$

where $R_{i}$ represents homogeneous or interfacial reactions when the equations are written in a volume-averaged form. In purely blocking transport problems, $R_{i}=0$ in the bulk; in battery electrodes, $R_{i}$ may represent insertion, plating, dissolution, or side reactions after appropriate averaging.

### 2.2. Poisson Coupling and Fixed, Regulated, and Induced Charge

The electrostatic potential is coupled to mobile and fixed charge by Poisson’s equation,

(5) $$-\nabla \cdot \left(\varepsilon \nabla \phi \right)=\rho_{f}\left(x\right)+e\sum_{i}z_{i}c_{i},$$

where ε is the dielectric permittivity and $\rho_{f}$ is a fixed charge density. In biological ion-channel equations, $\rho_{f}$ represents charged residues lining the pore. In energy-storage nanomaterials, analogous terms may arise from surface functional groups, ion-exchange sites, dopants, defects, charged polymer backbones, or immobile charges in interfacial layers. A second kind of charge is induced rather than prescribed, since jumps in the dielectric coefficient at a wall or protein/electrolyte boundary create polarization or dielectric-boundary charge, and the force from this induced charge can be large in narrow pores [19]. Mathematically, fixed or induced charge can produce internal layers, selectivity, nonlinear current–voltage response, and changes in the sign or magnitude of individual fluxes.

When free interfacial charge is represented by a surface density $\sigma_{s}$, the general electrostatic jump condition is

(6) $$n\cdot \left(D_{s}-D_{e}\right)=\sigma_{s},\;D_{\alpha }=-\varepsilon_{\alpha }\nabla \phi_{\alpha },$$

where n points from the electrolyte phase e into the adjoining solid phase s. If the electric displacement in the solid is neglected, Equation (6) reduces to

(7) $$n\cdot \left(\varepsilon_{e}\nabla \phi_{e}\right)=\sigma_{s}.$$

Writing the condition as a displacement jump avoids sign ambiguity and distinguishes a physical surface charge from a volumetric thin-layer approximation.

The surface charge need not be prescribed independently of the local state. For ionizable or specifically adsorbing surfaces, a charge-regulation condition has the schematic form

(8) $$\sigma_{s}=\sigma_{s}\left(\phi_{s},\left\{a_{i}\right\},T,\mathrm{pH}\right),$$

where $a_{i}$ denotes the activity of species *i*. Fixed-charge and charge-regulating closures should therefore be treated as different closures and compared when the surface chemistry is uncertain.

When a spatially varying permittivity is used in Equation (5), continuum dielectric polarization is already represented through the displacement field. An equivalent dielectric-boundary charge should not be introduced as an independent source unless the formulation explicitly separates free and bound charge and shows that no contribution is counted twice.

Equations (1)–(5) form the classical Poisson–Nernst–Planck system. The system is nonlinear because the electrostatic field depends on the concentrations and the concentrations are transported by the field. The nonlinearity is enhanced in the presence of narrow geometries, high-voltage regimes, concentrated electrolytes, or strong surface charges.

### 2.3. Current Conservation and Continuum-Variable Interpretation

A Poisson–Nernst–Planck calculation should also be checked as an electrodynamic current calculation. The mobile ionic charge density and ionic current density are

(9) $$\rho_{\mathrm{ion}}=e\sum_{i}z_{i}c_{i},\;J_{\mathrm{ion}}=e\sum_{i}z_{i}J_{i}.$$

Using Equation (4), the ionic charge balance is

(10) $$\frac{\partial \rho_{\mathrm{ion}}}{\partial t}+\nabla \cdot J_{\mathrm{ion}}=e\sum_{i}z_{i}R_{i}.$$

For a nonreactive electrolyte or for homogeneous reactions satisfying $\sum_{i}z_{i}R_{i}=0$, the right-hand side vanishes. In a reactive porous electrode, the ionic charge source must be balanced by the corresponding faradaic or electronic charge transfer.

When all free-charge carriers and charge-transfer pathways are represented consistently, let $\rho_{\mathrm{free}}$ and $J_{\mathrm{free}}$ denote the total free-charge density and free-current density. Local charge conservation and Gauss’s law then give

(11) $$\frac{\partial \rho_{\mathrm{free}}}{\partial t}+\nabla \cdot J_{\mathrm{free}}=0,\;\nabla \cdot J_{\mathrm{tot}}=0,\;J_{\mathrm{tot}}=J_{\mathrm{free}}+\frac{\partial D}{\partial t},\;D=\varepsilon E=-\varepsilon \nabla \phi .$$

In a one-dimensional series geometry, the complete Maxwell current must be independent of position. At a fully relaxed steady state, ∂D/∂t=0, and the measured current is the corresponding steady free current. During a voltage step, concentration change, or impedance perturbation, the displacement-current contribution is generally nonzero. Its time integral gives the change in electric displacement, or, equivalently, the associated stored charge.

Transient total-current consistency should be checked when the charging process is simulated. A sequence of independently computed steady states should instead be checked using steady free-current continuity, mass balance, and charge balance [20,21,22].

For energy-storage descriptions, this distinction is familiar from circuit representations in which capacitive double-layer and interphase currents are coupled with Faradaic, ionic, and electronic currents. A pore-scale, homogenized, or learning-based approximation should preserve the same complete current across series elements under a common transient protocol. Numerically, one can monitor the spatial variation of $J_{\mathrm{tot}}$, the charge accumulated in each element, and the agreement between the integrated charging current and the change in stored charge. For a one-dimensional calculation with nonzero current, a dimensionless residual is

(12) $$R_{J}=\frac{\max_{x}\left|J_{\mathrm{tot}}\left(x\right)-\langle J_{\mathrm{tot}}\rangle \right|}{\max_{x}\left|J_{\mathrm{tot}}\left(x\right)\right|},$$

where $\langle J_{\mathrm{tot}}\rangle$ is the spatial mean. A different scale must be stated when the denominator vanishes.

The discreteness of ions does not by itself invalidate continuum equations. Concentrations, charge densities, and fluxes can be defined as ensemble-averaged, coarse-grained, or probabilistic quantities associated with many-particle dynamics. Derivations of Poisson–Nernst–Planck-type equations from Langevin descriptions make this interpretation explicit [23,24]. However, a closed continuum transport description still depends on constitutive assumptions concerning mobilities, correlations, dielectric response, boundary conditions, and unresolved degrees of freedom. It may therefore be a poor physical approximation if it uses a dilute closure in a crowded pore, a spatially averaged surface charge where charge patches dominate, or a sharp boundary where a structured interphase is experimentally resolved.

### 2.4. Dimensionless Groups and Transport Regimes

A useful dimensionless parameter is the Debye ratio

(13) $$\epsilon_{D}=\frac{\lambda_{D}}{L},\;\lambda_{D}={\left(\frac{\varepsilon k_{B}T}{e^{2}\sum_{i}z_{i}^{2}n_{i}^{*}}\right)}^{1/2},$$

where *L* is the device, pore, or reference length scale and $n_{i}^{*}$ is a reference number density. If a molar concentration $C_{i}^{*}$ is used, then $n_{i}^{*}=N_{A}C_{i}^{*}$. When $\epsilon_{D}\ll 1$, thin-double-layer approximations and electroneutral bulk approximations may be appropriate. When $\epsilon_{D}=O\left(1\right)$, diffuse charge fills an appreciable fraction of the pore, and full or modified Poisson–Nernst–Planck equations may be required. Equation (13) gives the dilute-solution, linear-response Debye length. In concentrated electrolytes, ionic liquids, or strongly correlated confined fluids, it should be treated as a nominal comparison scale unless a generalized screening length has been derived from the thermodynamic response and correlations represented by the selected constitutive relation.

Other important dimensionless quantities include the dimensionless voltage $e\Delta \phi /k_{B}T$ and the local occupied-volume fraction

(14) $$\theta =\sum_{i}v_{i}n_{i}=N_{A}\sum_{i}v_{i}C_{i},$$

where $v_{i}$ is the effective molecular volume of species *i*, $n_{i}$ is its number density, and $C_{i}$ is its molar concentration. Finite-size corrections become important when θ is no longer small. Other useful quantities include a Péclet number comparing advection and diffusion, a Damköhler number $\mathrm{Da}=\tau_{\mathrm{transport}}/\tau_{\mathrm{reaction}}$, a Dukhin number comparing surface and bulk conduction, and tortuosity/porosity parameters in electrode-scale descriptions. These quantities should be reported whenever possible because they help determine whether the selected equations resolve the relevant physical regime.

Table 2 summarizes the dimensionless quantities used to report these transport regimes. Table 3 defines the quantities used in the two model checks and in the validation discussion.

### 2.5. Modified Poisson–Nernst–Planck Equations and Finite-Size Effects

The simplest classical Poisson–Nernst–Planck formulation is a mean-field, dilute-solution theory. It neglects finite ion size, ion–ion correlations, detailed solvation, and nonlocal molecular polarization. A spatially varying continuum permittivity can be included through Equation (5), but this does not reproduce molecular solvent layering or dielectric response by itself.

In a narrow pore or interphase, hydrated or solvated ions may occupy an appreciable fraction of the accessible volume. For an equal-site-volume lattice-gas approximation, Equation (14) reduces to $\theta =v\sum_{j}n_{j}$, and the excluded-volume contribution is

(15) $$\mu_{i}^{\mathrm{ex}}=-k_{B}T\ln\left(1-\theta \right),$$

which penalizes crowding as the unoccupied fraction tends to zero. This is a local equal-size closure. Unequal mobile-ion sizes, different solvation shells, neutral solvent occupancy, and pore-lining groups generally require species-dependent or nonlocal descriptions.

For energy storage materials, finite-size corrections can affect ion exclusion, partitioning, differential capacitance, access to active sites, and transport through confined electrolytes. The correct size variable is not always a bare ionic radius. It may represent a solvated ion, an ion aggregate, a lattice site, or the volume excluded by a functional group or polymer backbone. A useful model should state which interpretation is used and should test its sensitivity.

Modified Poisson–Nernst–Planck, Poisson–Fermi, classical density functional, and energetic-variational descriptions provide different ways to include crowding, activity, and correlations [25,26,27,28,29,30]. No one closure is uniformly best. A local lattice model is inexpensive and may be suitable for a pore several molecular diameters wide. Nonlocal or molecular methods become more important when layering, dielectric response, and detailed coordination determine the observable.

### 2.6. Concentrated Electrolytes, Boundary Layers, and Model Comparison

A concentrated electrolyte cannot generally be described by an ideal dilute chemical potential and diagonal mobility. Activity, cross-coupled transport, solvent or void occupancy, ion association, and concentration-dependent transport coefficients may all matter. Water-in-salt and hydrate-melt electrolytes provide clear examples because the solvent is no longer a passive majority component and interfacial structure changes with concentration [31,32,33,34,35,36,37].

Concentrated-solution theory and charge-resolved electrodiffusion address different questions. Onsager or Stefan–Maxwell relations describe coupled species transport and nonideal thermodynamics, usually under a bulk electroneutrality approximation. A Poisson–Nernst–Planck or Poisson–Fermi description resolves local charge separation through Poisson coupling. In many battery electrodes, the first description is suitable in the bulk electrolyte and the second is needed only in a narrow pore, an interphase, or a solid-state space-charge region. A multiscale model may therefore use both, with matched fluxes and electrochemical potentials at their common boundary.

Boundary layers arise when $\epsilon_{D}\ll 1$ and local charge is confined near an interface, pore entrance, or change in fixed charge. They can be resolved directly or replaced by effective boundary conditions. The choice depends on the target observable. A cell voltage may be insensitive to the detailed profile but sensitive to its integrated resistance or capacitance. A local spectroscopy or microscopy measurement may require the profile itself. Singular perturbation and homogenization provide systematic ways to derive such reduced laws when scale separation is present [6,38,39].

Table 4 gives a direct comparison of the main model families discussed in this Perspective.

DFN and concentrated-solution theory are therefore not replaced by charge-resolved models. They remain the practical basis for device prediction. The added value of Poisson–Nernst–Planck, modified Poisson–Nernst–Planck, and Poisson–Fermi descriptions appears when local electroneutrality is not valid or when fixed charge and confinement control transport. They can resolve ion partitioning, space-charge barriers, differential capacitance, selectivity, rectification, concentration buffering, and the response to a spatial charge pattern. These outputs can be reduced to an effective interfacial resistance, capacitance, transfer number, pore conductivity, or boundary condition and then passed to a porous-electrode model.

## 3. Energy Storage Cases and Cross-Scale Model Handoffs

The appropriate model depends on the system, the observable, and the scale at which a decision is made. This section gives representative cases for three important energy storage classes. The examples are based on the literature and do not constitute new validation data from the present authors.

### 3.1. Liquid-Electrolyte Batteries

For a conventional lithium-ion cell, a DFN or related porous-electrode model is a natural starting point because it connects solid diffusion, concentrated electrolyte transport, charge conservation, and interfacial kinetics to measured voltage. Its predictive value depends strongly on parameter quality. Chen et al. reported a broad experimental parameter set for a commercial 21700 cell and tested the resulting P2D model against discharge and relaxation profiles [8]. Zülke et al. used a separately parameterized DFN model to predict galvanostatic and drive-cycle responses of a commercial high-energy cell [9]. These studies show what a device model can do when geometry, thermodynamics, kinetics, and transport are measured carefully.

A local charge-resolved model becomes useful when the question concerns a narrow charged pore, a coating, or a structured interphase that is not represented by bulk electroneutral transport. Its output should not remain isolated. A pore calculation can provide an ion-partition coefficient, concentration-dependent conductance, differential capacitance, or effective interface resistance for the DFN model. Operando concentration measurements offer a second level of validation. X-ray fluorescence measurements of solution-phase concentration profiles in a positive electrode show that electrolyte transport can be tested against a spatial field rather than only against terminal voltage [10].

### 3.2. All-Solid-State Batteries

Solid electrolytes require defect transport, electrochemical potential matching, and interface models that may differ from liquid double-layer theory. Space-charge effects can change carrier concentration and interfacial resistance, but their importance is dependent on the material and interface [40]. Swift et al. combined a Poisson–Fermi–Dirac description with density-functional calculations to represent electronic band bending and ionic defect redistribution at solid-state interfaces and to examine interlayer thickness [12]. Wang et al. directly observed charge-density variations at a LiCoO_2_/Li_6_PS_5_Cl interface with in situ differential phase-contrast scanning transmission electron microscopy and compared the observations with finite-element calculations and impedance measurements [11].

These studies suggest a clear handoff. The local interface model resolves defect and potential profiles. Its integrated resistance, capacitance, and field-dependent transport law then enter an electrode or cell model. The interface description should be tested against local charge or potential data when available and against impedance and rate response at the cell level.

### 3.3. Nanoporous Supercapacitors

Supercapacitors provide a direct setting in which ion packing, electrode polarization, pore structure, and charging time are all central. Constant-potential molecular simulations of realistic carbide-derived carbon with a polarizable electrolyte have produced integral capacitances in quantitative agreement with experimental findings [13]. In conductive metal–organic framework electrodes, molecular simulation, material synthesis, and multiscale modeling have been combined to connect solvent content with capacitance and charging speed [14]. A pore-network description has also been checked against simplified Poisson–Nernst–Planck calculations and experimental data for porous carbon charging [15].

Here, the model handoff can proceed from molecular ion occupancy and free energy to a pore-level capacitance and mobility, then to a network charging time or transmission-line law, and finally to device-level power and energy. Agreement in terminal capacitance alone is not sufficient. Charging time, voltage dependence, ion occupancy, and pore-size sensitivity provide additional checks.

### 3.4. Structured Comparison of Applications

Table 5 gives a structured comparison. Each row states the local question, model sequence, measurable evidence, and quantity passed to the coarser model.

**Figure 2:**
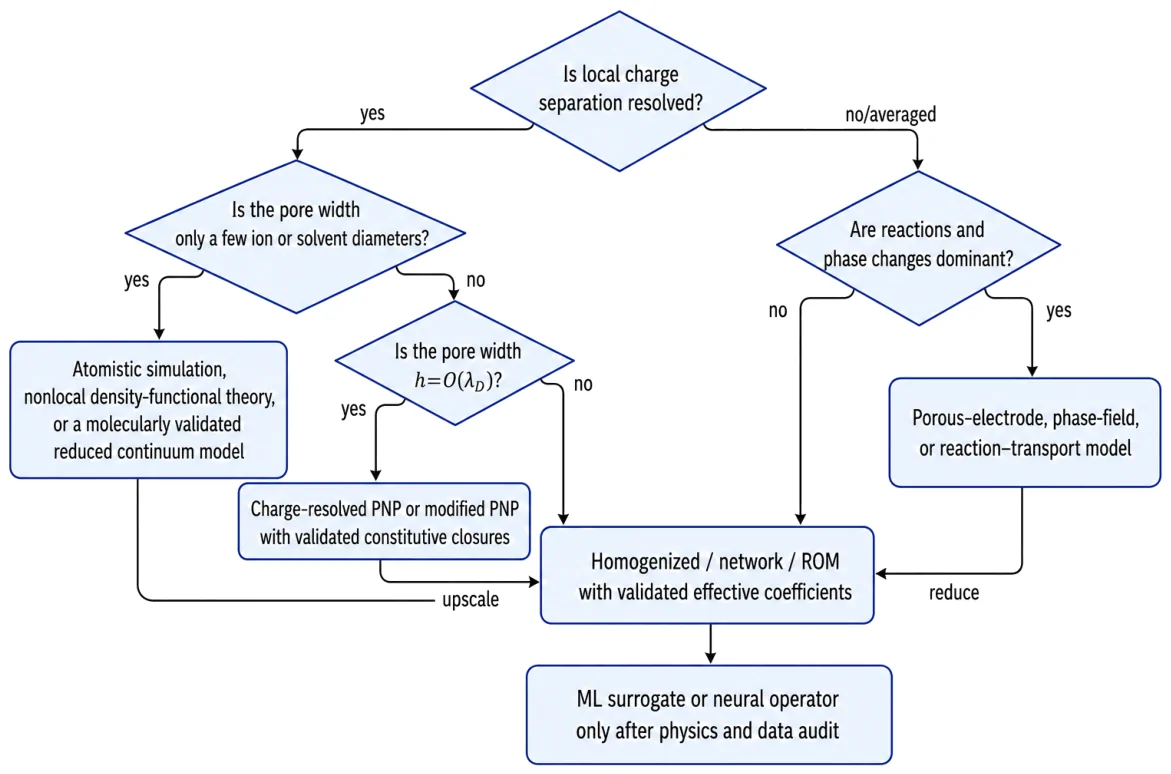
A practical selection procedure for charged nanostructured transport. Charge-resolved or modified Poisson–Nernst–Planck equations are suitable when local charge separation matters and continuum fields remain meaningful. Atomistic or nonlocal descriptions are needed when the pore is only a few molecular diameters wide. Reaction-transport, porous-electrode, or phase-field models become central when reactions, phase change, or evolving morphology dominate. Homogenized, network, reduced-order, and learning-based descriptions should be introduced only after the local regime and the quantities passed between scales have been identified. Diamonds denote decision points, whereas rectangular boxes denote model classes or model-reduction and transfer steps.

The practical selection procedure is shown in Figure 2. Charge-resolved continuum equations are most useful when local charge separation matters and the pore remains large enough for continuum fields to have meaning. Molecular or nonlocal descriptions are preferred when only a few molecular layers fit in the pore. Porous-electrode, phase-field, and reaction-transport models become central when reactions, phase change, or evolving morphology dominate. In every case, the selected local model should return a quantity that can be measured or passed to the next scale.

## 4. Transferable Electrodiffusion Ideas and Limits of the Ion-Channel Analogy

Ion channels are not chemical models of batteries or supercapacitors. They are useful here because the mathematical literature treats narrow geometry, fixed charge, boundary layers, unequal ion sizes, and boundary-driven flux as separate variables. This article uses these ideas as diagnostic tools as opposed to organizing the energy storage discussion around a biological analogy.

One transferable idea is to represent charge as a spatial distribution. A single fitted conductivity cannot distinguish a uniformly charged pore from a localized charge patch, even though the two cases may have different ion partitioning, rectification, and current response. In nanomaterials, the relevant sources may be surface functional groups, dopants, lattice defects, charged polymer backbones, or reaction products. Fixed, regulated, and dielectric-induced charge should be modeled with different closures.

A second idea is to separate thin layers from bulk transport. Steady Poisson–Nernst–Planck analysis has shown how slow regions, fast boundary layers, and matching conditions arise when the Debye scale is small [38,39]. In an energy storage model, the same mathematical structure can support an effective boundary condition or interfacial impedance rather than a resolved layer. This reduction is useful only when the scale separation is checked.

A third idea is to audit a finite-size approximation by comparing orders rather than by reporting one corrected current curve. Higher-order terms can become important when a lower-order coefficient is small by symmetry or cancellation [42,43]. This does not automatically mean that the full correction is large. The first calculation in Section 5 illustrates the distinction.

A fourth idea is to preserve complete current and mass balance. A pore, interphase, and porous electrode connected in series must carry a consistent total current under the same transient protocol. The displacement-current contribution matters during charging, while steady calculations require consistent free-current continuity. A reduced model or surrogate should be checked against these balances independently of the equations used in training or fitting.

The limits of the analogy are equally important. Energy storage devices include electron transport, electrochemical reactions, phase transformations, mechanics, interphase growth, thermal effects, and manufacturing-dependent microstructure. These effects are not contained in an ion-channel model. Charge-resolved electrodiffusion should therefore be used as a local component when the regime requires it, then coupled to the appropriate reaction, solid diffusion, and device equations. The goal is not to apply an ion-channel equation unchanged to a battery. The goal is to use well-defined mathematical checks where a narrow charged region controls a measurable energy storage response.

## 5. Two Model-Selection Checks

The calculations in this section are deliberately simple. They do not introduce a new battery chemistry or claim experimental validation. They answer two model-selection questions. The first asks when a first-order finite-size reduction is a reliable summary of a modified electrodiffusion model. The second asks when local electroneutrality is an inaccurate approximation in a charged pore and how finite packing changes the screening response.

### 5.1. Audit of a Finite-Size Reduction

Consider the truncated current expansion

(16) $$I\left(\hat{V};d\right)=I_{0}\left(\hat{V}\right)+dI_{1}\left(\hat{V}\right)+d^{2}I_{2}\left(\hat{V}\right)+o\left(d^{2}\right),\;\hat{V}=\frac{eV}{k_{B}T},$$

where $I_{0}$ is the classical Poisson–Nernst–Planck current and $I_{1}$ and $I_{2}$ are finite-size corrections. The dimensionless cation size is *d*, the anion size is λd, and λ is their size ratio. The parameter *d* is an expansion parameter rather than a dimensional ion diameter.

The useful question is not whether the second-order term is ever larger than the first. A small first-order coefficient can make this occur by cancellation. The relevant checks are

(17) $$R_{1}=\frac{∥dI_{1}{∥}_{2}}{∥I_{0}{∥}_{2}},\;R_{2}=\frac{∥d^{2}I_{2}{∥}_{2}}{∥dI_{1}{∥}_{2}},\;R_{\mathrm{corr}}=\frac{∥dI_{1}+d^{2}I_{2}{∥}_{2}}{∥I_{0}{∥}_{2}},$$

computed over a stated voltage interval. The ratio $R_{2}$ tests first-order dominance, while $R_{\mathrm{corr}}$ tests the total correction relative to the classical current. Thus, $R_{2}>1$ can occur even when the full finite-size correction remains small.

When a full modified Poisson–Nernst–Planck solution is available, the truncation should also be checked directly through

(18) $$E_{\mathrm{trunc}}=\frac{∥I_{\mathrm{full}}-I_{0}-dI_{1}-d^{2}I_{2}{∥}_{2}}{∥I_{\mathrm{full}}{∥}_{2}}.$$

**Figure 3:**
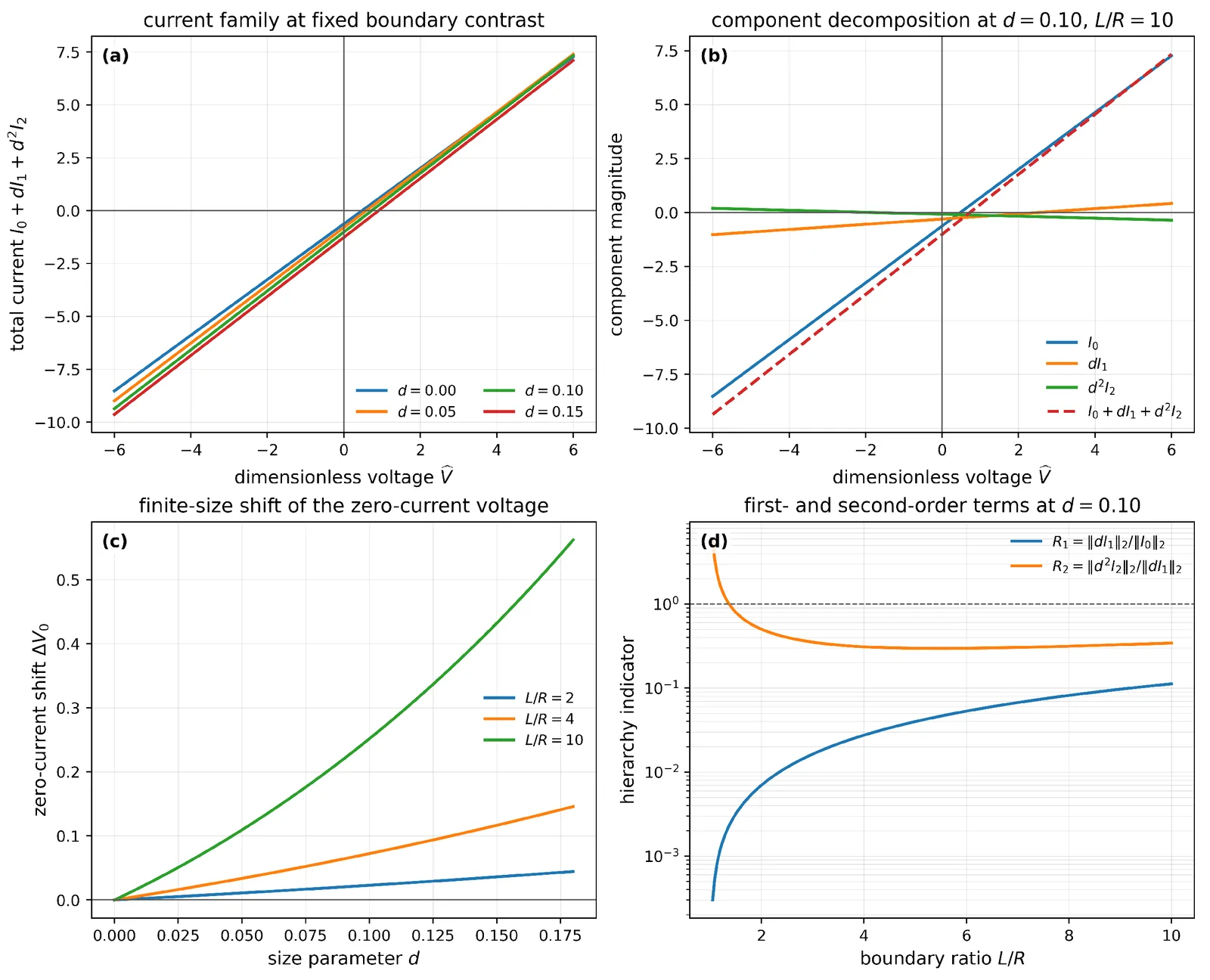
Audit of a finite-size Poisson–Nernst–Planck expansion. Panel (**a**) gives corrected current families for several values of *d*. Panel (**b**) separates $I_{0}$, $dI_{1}$, and $d^{2}I_{2}$. Panel (**c**) reports the finite-size shift of the zero-current voltage. Panel (**d**) compares the first- and second-order contributions. The overlap of several curves is intentional and reflects their close numerical agreement. The gray horizontal and vertical lines in panels (**a**) and (**b**) are zero-reference lines. The dashed line $R_{2}=1$ in panel (**d**) is an order-comparison marker and is not a stand-alone failure boundary.

Figure 3 shows the audit for a two-species test problem with unequal diffusion and unequal size. Panel (a) shows how the corrected current changes with *d*. Panel (b) separates the three terms. Panel (c) shows the shift in zero-current voltage. Panel (d) shows that the relative order comparison can deteriorate near balanced boundary conditions because the first-order term becomes small. The scientific purpose is therefore to identify when a reduced finite-size law can be passed to a coarser model without hiding a cancellation.

### 5.2. Audit of Electroneutrality and Charge Screening

The second check uses a symmetric monovalent electrolyte in a one-dimensional charged pore. With $\psi =e\phi /k_{B}T$, x=X/L, $\epsilon_{D}=\lambda_{D}/L$, and a dimensionless fixed charge $q_{f}$, the equilibrium equation is

(19) $$-2\epsilon_{D}^{2}\psi^{\prime \prime }=q_{f}\left(x\right)+c_{+}\left(\psi \right)-c_{-}\left(\psi \right),\;\psi \left(0\right)=\psi \left(1\right)=0,$$

with the equal-size Bikerman closure

(20) $$c_{\pm }\left(\psi \right)=\frac{\exp(\mp \psi )}{1+2\nu (\cosh\psi -1)},\;0\leq 2\nu <1.$$

The parameter ν is the bulk lattice occupancy of one species. The prescribed central charge is

(21) $$q_{f}\left(x\right)=\frac{q_{0}}{2}\left[\tanh\;\left(\frac{x-0.35}{0.015}\right)-\tanh\;\left(\frac{x-0.65}{0.015}\right)\right].$$

The global screening residual and a maximum-norm companion are

(22) $$E_{Q}=\frac{∥q_{f}+c_{+}-c_{-}{∥}_{L^{2}\left(0,1\right)}}{∥q_{f}{∥}_{L^{2}\left(0,1\right)}},\;E_{Q,\infty }=\frac{∥q_{f}+c_{+}-c_{-}{∥}_{L^{\infty }\left(0,1\right)}}{∥q_{f}{∥}_{L^{\infty }\left(0,1\right)}}.$$

The first measures the integrated charge defect. The second detects a narrow residual that may be hidden in the global norm. The steric effect is summarized by

(23) $$A_{\psi }\left(\nu \right)=\frac{\int_{0}^{1}\left|\psi \left(x;\nu \right)\right|\;dx}{\int_{0}^{1}\left|\psi \left(x;0\right)\right|\;dx}.$$

**Figure 4:**
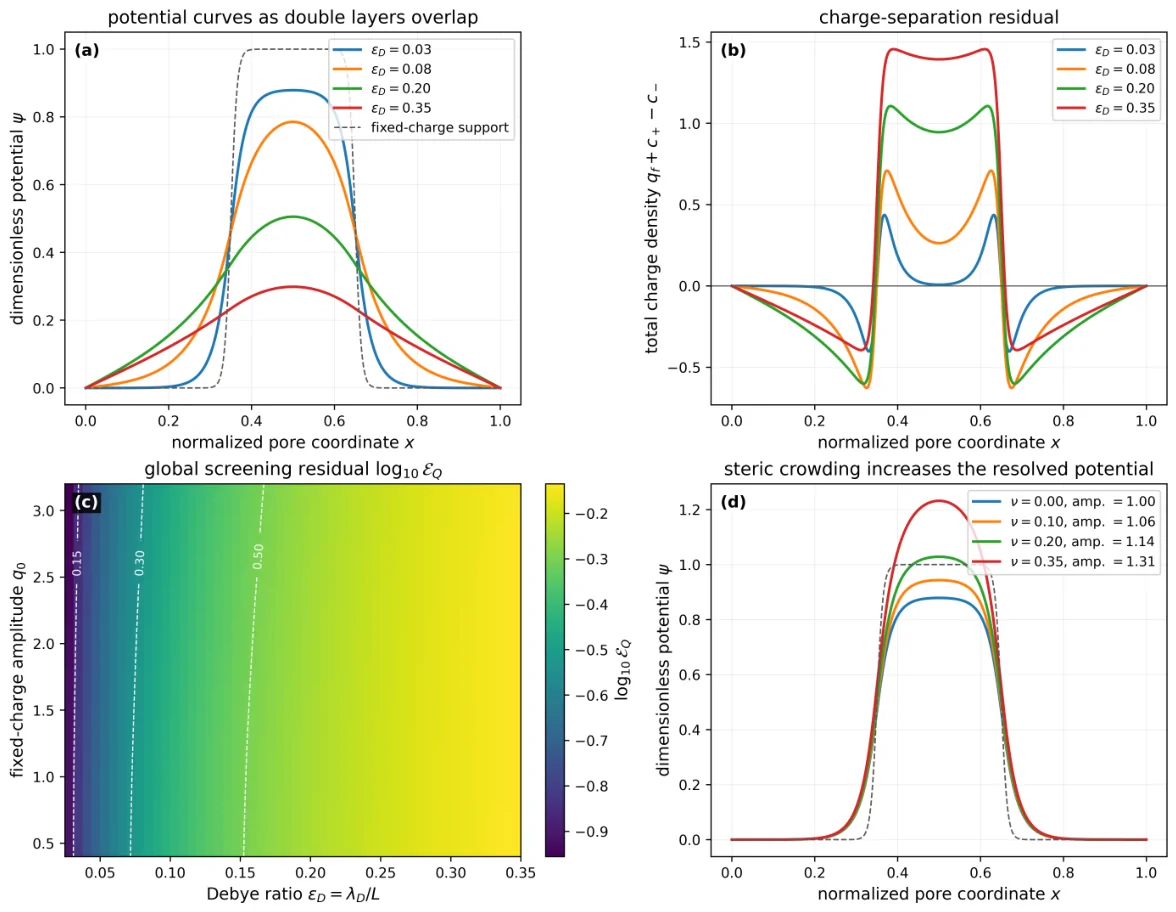
Equilibrium screening audit for a one-dimensional charged pore. Panel (**a**) shows potential profiles for several Debye ratios. Panel (**b**) shows the total charge density. Panel (**c**) gives the global screening residual in the $\left(\epsilon_{D},q_{0}\right)$ plane. Panel (**d**) shows how finite packing changes the potential required to screen the prescribed charge. The overlap of some colored curves is intentional and indicates similar profiles for the corresponding parameter values. The gray dashed curves in panels (**a**) and (**d**) mark the prescribed fixed-charge support, while the dashed contours in panel (**c**) indicate labeled screening-residual levels.

Figure 4 answers the second model question. Small $\epsilon_{D}$ produces a nearly screened interior with residual charge concentrated near transitions. As $\epsilon_{D}$ grows, charge separation spreads across more of the pore and a bulk electroneutral approximation becomes less accurate. Increasing ν limits counterion packing, and so a larger potential is needed to screen the same fixed charge. The useful output for a coarser model is not the full profile in every case. It may be an effective capacitance, partition law, or interfacial resistance derived from that profile.

Table 6 gives only a conditional physical reading of the nondimensional parameters. It does not identify a particular pore chemistry.

#### Numerical Methods and Limits

Figure 3 was generated from the closed-form finite-size expansion in Python 3.13.5 using NumPy 2.3.5 and Matplotlib 3.10.8. The current curves use 401 equally spaced voltages on [−6,6], and the norm ratios use exact continuous $L^{2}$ norms of the affine current terms. Figure 4 was computed with second-order centered finite differences and a damped Newton iteration. Curves use 1201 nodes and an infinity-norm nonlinear residual tolerance of $10^{-10}$. The screening map uses 401 nodes, continuation in $q_{0}$, and a residual tolerance of $2\times 10^{-8}$. A mesh check with 2401 nodes gave a largest maximum-norm potential difference of $1.49\times 10^{-5}$ and a largest absolute change in $E_{Q}$ of $3.61\times 10^{-6}$ over five test cases.

Both checks have clear limits. They use one-dimensional geometry, effective permittivity, smooth boundary data, and a prescribed volume-charge region. They do not resolve solvent layering, charge regulation, patchy chemistry, pore networks, reactions, or mechanics. Their value is to show how a model assumption can be tested before it is used in a larger energy storage calculation.

## 6. Machine Learning and Reduced Models

No machine-learning model is trained or assessed in this article. The purpose of this section is to distinguish several recent uses of learning in energy storage and to state what evidence is needed before a learned model is used for ion transport.

One use is property prediction from material and processing data. Minakshi et al. used several learning methods to relate biomass source, pore properties, processing conditions, and electrochemical settings to the specific capacitance of aqueous symmetric supercapacitors. The model was then discussed together with a synthesized porous carbon device [44]. This is a useful example of materials and process prediction, but it is not a solver for local concentration, potential, or ionic flux.

A second use is experiment-guided electrolyte optimization. Yan et al. combined automated high-throughput measurements with regression and uncertainty estimates to optimize the ionic conductivity of non-aqueous battery electrolytes [45]. Here, the learned output is a measured bulk property. The main requirements are a well-defined composition space, representative experiments, and uncertainty outside the sampled region.

A third use is a surrogate for a physical battery model. Neural operators and related models can learn a parameter-to-solution map and accelerate repeated simulation. Recent work has compared operator-learning methods for lithium-ion battery equations with inputs that include current, particle size, and diffusivity [46]. Such surrogates should be compared with an independently run solver and with experiments used neither in training nor in parameter fitting.

For the charge-resolved equations discussed in this Perspective, suitable learning targets include a pore conductivity, an interfacial resistance, a closure relation, a low-dimensional observable, or a parameter-to-solution map. These targets are easier to validate than unrestricted field prediction. Inputs should state the charge distribution, geometry, concentration range, voltage range, occupied-volume fraction, and reaction time-scale ratio when these variables affect the result.

Table 7 summarizes the minimum checks.

Physics-informed neural networks impose equations and data through a loss function [47,48]. For stiff electrodiffusion problems, small training loss does not guarantee that narrow boundary layers are resolved. Neural operators, including DeepONet and Fourier neural operator families, are suitable when the input is itself a function, such as a charge distribution, pore shape, or cycling protocol [49,50]. Reduced-order methods provide an intermediate option when a small set of physically meaningful variables can be retained. A useful division of labor is for analysis and simulation to identify the variables and limits, while learning approximates only the expensive map or closure that remains.

## 7. Experimental and Simulation Validation

The present article does not claim experimental validation of its two generic calculations. Instead, it states how a model of an energy storage system should be verified with published or future measurements. Validation should match the variable resolved by the model. A cell model is tested with cell observables. A pore or interface model requires a local or integrated observable that is sensitive to the local physics.

For a liquid-electrolyte battery, terminal voltage is necessary but not sufficient. Relaxation, rate response, EIS, and concentration profiles constrain different parameter combinations. The parameterization and prediction studies in [8,9] show how a DFN model can be tested across operating protocols. Operando X-ray fluorescence provides a direct electrolyte concentration profile for transport validation [10].

For an all-solid-state interface, useful observables include interfacial resistance, capacitance, charge or potential profiles, and rate response. In situ differential phase-contrast microscopy has provided direct charge-density evidence at a working cathode–solid electrolyte interface, together with impedance and finite-element comparison [11]. A Poisson–Fermi or defect model should reproduce the sign, width, and operating-condition dependence of the space-charge region before its integrated resistance is used in a cell model.

For a nanoporous supercapacitor, capacitance should be checked together with charging time, voltage dependence, and, when available, ion occupancy or structural measurements. Molecular simulations that reproduce experimental capacitance and combined simulation–experiment studies of conductive MOF electrodes show how local ion organization can be connected to device response [13,14]. Pore-network predictions can be compared with simplified electrodiffusion calculations and measured relaxation or impedance [15].

Table 8 summarizes these routes.

A useful comparative test for fixed or regulated charge is a controlled change in external concentration. A strongly charged pore may change its counterion content, conductance, or capacitance less than a weakly charged reference when the bath concentration is changed. This buffering response is measurable, but it is not unique evidence of fixed charge. Charge regulation, concentration-dependent mobility, changing pore access, and interfacial reactions can produce similar trends. The comparison is strongest when charged and reference samples have matched geometry and when several observables are measured.

Parameter estimation should be treated as an inverse problem. Diffusion coefficients, transfer numbers, exchange-current densities, tortuosity, surface charge, packing parameters, and interfacial resistance may not be identifiable from one voltage curve. Multiple protocols, sensitivity analysis, and uncertainty intervals are needed to determine which parameters are informed by the data.

**Figure 5:**
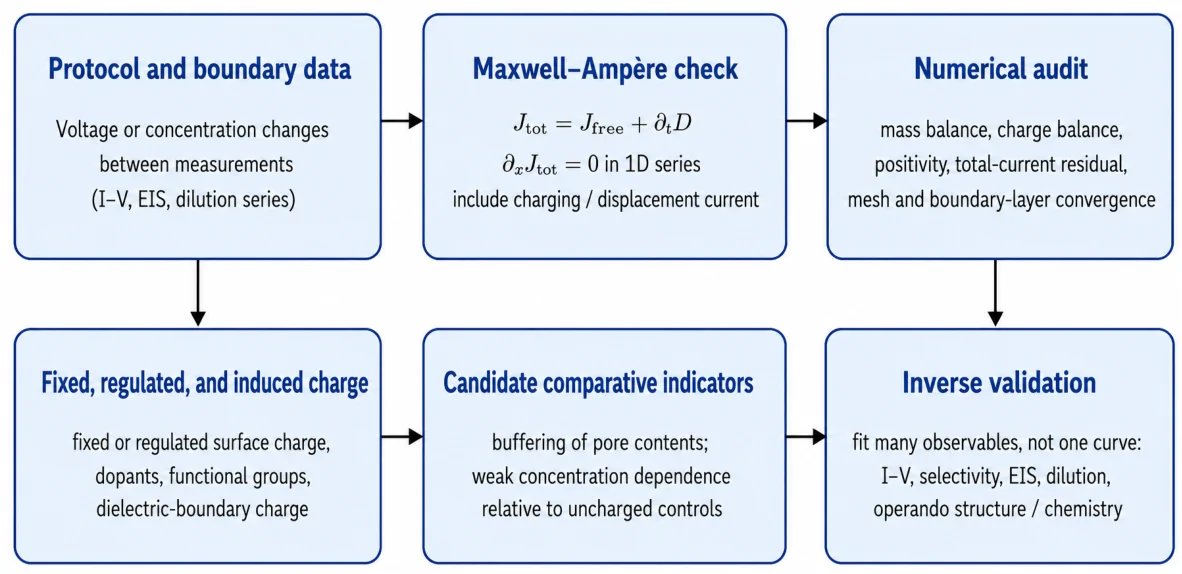
Checks for charge-aware ion transport descriptions. The upper branch tests whether free and displacement current form a spatially consistent total current and whether mass, charge, and numerical convergence checks are satisfied. The lower branch connects fixed, regulated, or induced charge to comparative observables and then tests those observables with more than one measurement type. Arrows indicate the direction of the validation sequence.

Figure 5 summarizes the validation sequence. The upper branch checks current and numerical consistency under a common protocol. The lower branch connects charge assumptions to comparative observables and then to inverse validation.

## 8. Challenges and Outlook

The main challenge is not a lack of transport models. It is the absence of clear, tested rules for moving between them. Several issues are especially important.

The first is cross-scale handoff. A molecular or pore calculation should return a defined quantity such as a free-energy profile, transfer number, pore conductivity, differential capacitance, or interface resistance. The uncertainty in that quantity should be carried into the electrode model. A local calculation that cannot be connected to an observable or a coarser equation has limited value for device prediction.

The second is interface definition. At nanometer scales, the charge plane, mass-density interface, dielectric dividing surface, closest ion approach, diffusion interface, and reaction plane may not coincide. A model should state which effective surface is used. Fixed charge, regulated charge, and dielectric polarization should not be inserted as independent sources when they describe the same physical contribution.

The third is regime identification. Pore width, nominal screening length, ion or solvent size, occupied-volume fraction, voltage, surface-to-bulk conduction, reaction time scale, and tortuosity should be reported whenever possible. These quantities determine whether electroneutral concentrated-solution transport, charge-resolved continuum equations, or molecular methods are appropriate.

The fourth is experimental identifiability. Voltage alone rarely determines all transport and interface parameters. Operando concentration profiles, impedance, microscopy, spectroscopy, and controlled perturbations should be combined so that competing explanations can be separated. Validation should include conditions different from those used in parameter fitting.

The fifth is numerical and learning-based reproducibility. Reference problems should report geometry, boundary conditions, nondimensional parameters, mesh and solver errors, conservation residuals, and code or sufficient algorithmic detail. Learned surrogates should be tested on changed charge distributions, geometries, and material classes rather than only on random samples from one distribution.

The most useful future studies will connect these five tasks in one chain. A local mechanism should be identified, represented at the smallest necessary scale, reduced to an effective law with uncertainty, inserted into an electrode model, and tested against both local and device observables.

## 9. Conclusions

Ion transport in energy storage nanomaterials spans molecular, pore, interfacial, electrode, and device scales. No single equation resolves all of them. DFN and concentrated-solution models remain central for battery and device prediction. Charge-resolved Poisson–Nernst–Planck, modified Poisson–Nernst–Planck, and Poisson–Fermi descriptions add local electric potential, ion partitioning, space charge, finite packing, and capacitance when electroneutral bulk assumptions are not adequate. Molecular simulation supplies interfacial structure and constitutive information, while pore-network and homogenized models connect local laws to electrode geometry.

The main point of this Perspective is a model-selection and handoff procedure. The model should be chosen from the observable and the regime, not from a fixed preference for one equation. Local outputs should be reduced to quantities that a coarser model can use. Validation should compare the correct variables and should include independent operating conditions and more than one observable.

Representative studies in liquid-electrolyte batteries, all-solid-state batteries, and nanoporous supercapacitors show that this program is practical. Device-scale voltage prediction, operando concentration profiles, direct imaging of interfacial charge, impedance, capacitance, and charging time provide complementary evidence. Recent work has also shown several distinct roles for machine learning, including material-property prediction, electrolyte optimization, and acceleration of physical battery models. These roles should not be treated as equivalent.

The two calculations retained in the article are model audits. They show how to detect cancellation in a finite-size reduction and how to test the loss of local electroneutrality in a charged pore. They are not presented as new device results. Their purpose is to illustrate the checks that should precede the use of a reduced local law in a larger energy storage model.

A reliable multiscale description should therefore state what is resolved, what is averaged, what is passed between scales, and which measurement can reject the model. This structure provides a clearer route from mathematical electrodiffusion to useful predictions for energy storage nanomaterials.

## Figures and Tables

**Table 1:** Main model classes used for ion transport in energy storage nanomaterials.

Scale or Role	Example Descriptions	Main Outputs	Useful Evidence	Main Limits
Electronic and atomistic	DFT, AIMD, classical MD	Migration barriers, solvation, adsorption, reaction pathways, local structure	Spectroscopy, diffraction, diffusion data, interfacial structure, thermodynamic limits	Limited length and time scales; sensitivity to functional or force field
Molecular to pore	Classical or modified Poisson–Nernst–Planck, Poisson–Fermi, density-functional transport	Potential, local charge, ion partitioning, selectivity, capacitance, current	Concentration dependence, capacitance, transference, local charge or potential, resolved simulations	Continuum closure may fail in molecular pores; parameters may be difficult to identify
Mesoscale	Pore networks, phase field, kinetic Monte Carlo, microstructure-resolved finite elements	Connectivity, tortuosity, moving interfaces, effective transport	Microscopy, tomography, morphology evolution, impedance	High parameter demand; a representative microstructure is required
Electrode and device	Porous-electrode theory, DFN or P2D, concentrated-solution transport, transmission lines	Voltage, concentration polarization, utilization, charge and discharge response	Voltage, relaxation, EIS, rate tests, operando concentration profiles	Nanoscale charge and interphase structure are averaged into effective laws
Interphase focused	Reaction–diffusion, moving boundary, defect transport, chemomechanics	Growth, leakage, resistance, stress, capacity loss	EIS, microscopy, spectroscopy, postmortem analysis	Composition, geometry, and reaction pathways may be non-unique
Learning and reduced	ROMs, PINNs, neural operators, graph models, Bayesian optimization	Acceleration, inverse estimation, screening, uncertainty estimates	Independent solvers, hold-out experiments, conservation tests, out-of-domain cases	Extrapolation risk, sampling bias, and incomplete physical constraints

**Table 2:** Dimensionless quantities useful for reporting ion transport regimes.

Quantity	Typical Form	Interpretation
Debye ratio	$\epsilon_{D}=\lambda_{D}/L$	Indicates whether diffuse charge is confined to thin layers or fills a substantial part of the pore or interphase.
Dimensionless voltage	$e\Delta \phi /k_{B}T$	Compares electrostatic driving with thermal energy. Large values may invalidate linear double-layer approximations.
Occupied-volume fraction	$\theta =\sum_{i}v_{i}n_{i}=N_{A}\sum_{i}v_{i}C_{i}$	Indicates when finite ion size and crowding require an explicit closure.
Dukhin number	Surface conduction divided by bulk conduction	Measures the importance of surface transport in charged pores and membranes.
Damköhler number	$\mathrm{Da}=\tau_{\mathrm{transport}}/\tau_{\mathrm{reaction}}$	Under this definition, values above one indicate reaction faster than transport.
Péclet number	Advective transport divided by diffusive transport	Relevant when flow, convection, or swelling-induced motion is present.
Tortuosity factor	Effective path length divided by geometric length	Represents microstructural obstruction in porous electrodes and separators.
Total-current residual	$R_{J}=\max_{x}|J_{\mathrm{tot}}-\langle J_{\mathrm{tot}}\rangle |/\max_{x}\left|J_{\mathrm{tot}}\right|$	Tests Maxwell–Ampere consistency and series current continuity.

**Table 3:** Notation used for charge, size, current, and screening checks.

Symbol	Meaning
θ	General local occupied-volume fraction, $\theta =\sum_{i}v_{i}n_{i}$.
*d*, λ	Finite-size expansion parameter and anion-to-cation size ratio in Section 5. The parameter *d* is not a physical length unless a normalization is stated.
ν	Lattice occupancy of one bulk ionic species in the screening calculation, $\nu =vn_{b}$. The total bulk ionic occupancy is $\theta_{b}=2\nu$.
$q_{f}$, $q_{0}$	Dimensionless fixed-charge density and its amplitude in the screening calculation.
$\epsilon_{D}$	Debye ratio $\lambda_{D}/L$.
$J_{\mathrm{ion}}$, $J_{\mathrm{free}}$, $J_{\mathrm{tot}}$	Ionic current, total free current, and Maxwell total current.
$R_{J}$	Spatial total-current residual in Equation (12).
$R_{1}$, $R_{2}$, $R_{\mathrm{corr}}$	First-order, order-comparison, and total finite-size correction ratios.
$E_{\mathrm{trunc}}$	Error of the second-order asymptotic truncation when a full modified Poisson–Nernst–Planck solution is available.
$E_{Q}$, $E_{Q,\infty }$	Global $L^{2}$ and maximum-norm charge-screening residuals.
$A_{\psi }$	Potential-amplification factor in the steric screening comparison.

**Table 4:** Direct comparison of model families for ion transport in energy storage materials.

Model Family	Main Resolved Output	Charge and Nonideality	Best Use	Main Limit and Handoff
DFN or porous-electrode model with concentrated-solution transport	Cell voltage, solid-state diffusion, electrolyte polarization, utilization, reaction current	Usually electroneutral bulk electrolyte with nonideal transport and effective interface laws	Electrode and cell prediction over charge, discharge, and drive cycles	Local double layers and interphase structure are averaged; accepts effective transport, resistance, capacitance, and kinetic laws
Classical Poisson–Nernst–Planck	Local concentrations, potential, ionic flux, space charge	Dilute mean-field chemical potential with Poisson-resolved mobile and fixed charge	Charged mesopores, dilute electrolytes, and resolved double-layer overlap	Misses crowding and correlations; can supply partition coefficients, pore conductance, and interface laws
Modified Poisson–Nernst–Planck or Poisson–Fermi	Local charge, ion packing, activity corrections, potential, flux	Finite-size or activity closure with resolved charge; Poisson–Fermi can include correlation and defect occupancy effects	Confined concentrated electrolytes and solid-state space-charge regions	Closure parameters require molecular or experimental support; passes resistance, capacitance, selectivity, and defect profiles upward
Molecular simulation	Solvation, adsorption, coordination, layering, diffusion, free energies	Explicit particles and an atomistic or coarse-grained interaction model	Subnanometer pores, electrolyte structure, interfacial chemistry, and constitutive data	Limited scales and force-field uncertainty; supplies free energies, mobilities, reaction hypotheses, and local structure
Pore-network or homogenized model	Effective conductivity, diffusivity, tortuosity, charging time, network flow	Local charge law may be retained in pore elements or averaged into coefficients	Electrode microstructure and the link from pores to continuum electrodes	Depends on representative geometry and local closures; supplies effective coefficients to device models

**Table 5:** Structured examples of charge-aware transport in energy storage and ion-selective materials.

Application	Local Question	Model Sequence	Measurable Evidence	Handoff to a Coarser Model
Liquid-electrolyte lithium-ion battery	Does a pore or interphase alter local concentration, transfer number, or resistance?	Molecular or modified Poisson–Nernst–Planck model, then homogenization, then DFN	Voltage and relaxation, EIS, rate response, operando concentration profiles [8,9,10]	Effective diffusivity, conductivity, transfer number, interface resistance, kinetic correction
All-solid-state battery	Does defect redistribution create a space-charge barrier at the cathode–electrolyte interface?	DFT and defect thermodynamics, Poisson–Fermi or space-charge model, then electrode model	Local charge or potential, EIS, interfacial resistance, rate response [11,12]	Interfacial resistance, capacitance, defect-dependent mobility, effective boundary law
Nanoporous carbon or MOF supercapacitor	How do pore size, ion packing, and electrode polarization control capacitance and charging time?	Constant-potential MD or modified Poisson–Nernst–Planck, then pore network or transmission line	Differential or integral capacitance, charging time, adsorption, NMR or structural data, EIS [13,14,15]	Pore capacitance, mobility, relaxation time, network conductance
Single-ion or charged polymer electrolyte	How does fixed backbone charge affect counterion partitioning and transference?	Molecular simulation or concentrated-solution theory, local charge-resolved model when needed, then homogenization	Conductivity, transference number, limiting current, spectroscopy, concentration dependence	Effective mobility matrix, partition coefficient, fixed-charge density, conductivity
SEI or CEI layer	Is the interphase a boundary condition or a resolved transport region?	Atomistic reaction model, reaction–diffusion or Poisson–Nernst–Planck layer, then porous-electrode degradation model	Growth, leakage current, EIS, spectroscopy, microscopy, capacity loss [41]	Thickness-dependent resistance, permeability, reaction rate, moving-boundary law

**Table 6:** Approximate physical reading of the nondimensional parameters used in the two model checks.

Quantity	Range Used	Conditional Dimensional Interpretation
*d*	0 for the classical baseline; 0.02–0.18 for the finite-size cases	Under a normalization $d=a/L_{\mathrm{ref}}$ with $L_{\mathrm{ref}}=5$ nm, the corresponding effective size is about 0.10–0.90 nm for the nonzero cases. A different normalization changes this conversion.
$\epsilon_{D}$	0.025–0.35	For an effective pore dimension of 5–10 nm, the nominal screening length is about 0.13–3.5 nm. The dilute Debye formula is only a comparison scale at high concentration.
$q_{0}$	0.4–3.2	For a 0.1 M monovalent electrolyte, the corresponding volume-charge scale is about $3.9\times 10^{6}$–$3.1\times 10^{7}$ C m^−3^. A slit-pore interpretation gives a surface-charge scale of about 0.010–0.077 C m^−2^.
ν	0–0.35	The total bulk lattice occupancy is 2ν=0–0.70. At ν=0.35, site volumes of 0.1–0.5 nm^3^ correspond to about 5.8–1.2 M per species.
$\hat{V}$	−6–6	At 298 K, this interval is approximately −154 to 154 mV.

**Table 7:** Proposed checks for learning-based ion transport models.

Check	Minimum Evidence to Report
Clear task definition	State whether the model predicts a material property, an effective coefficient, a field, a current, or a device response.
Independent reference	Compare with an independently run physical solver or with measurements not used to construct the training data.
Separated evaluation sets	Distinguish interpolation, changed operating conditions, changed geometry or charge, and changed material class.
Physical admissibility	Check positivity, boundary conditions, mass balance, charge balance, and total current independently of the training loss.
Observable accuracy	Report errors in currents, voltage, capacitance, impedance features, or other measured quantities in addition to field errors.
Uncertainty and failure cases	Give calibrated uncertainty where possible and identify cases outside the training domain.
Computational value	Compare accuracy and wall-clock cost with those of the physical model that the surrogate replaces.

**Table 8:** Validation matrix for the model families discussed in this Perspective.

Model Target	Primary Comparison	Additional Test	Failure That the Test Can Reveal
DFN or porous-electrode response	Voltage under several currents and relaxation periods	EIS and operando electrolyte concentration	Compensating parameter errors that fit voltage but misrepresent transport
Charge-resolved liquid pore or interphase	Conductance, capacitance, transfer number, concentration dependence	Matched charged and weakly charged samples, local concentration or potential when available	Incorrect fixed charge, access resistance, or finite-size closure
Solid-state space-charge model	Local charge or potential profile and interfacial resistance	Bias dependence, temperature dependence, and rate response	Wrong defect chemistry, interface position, or dielectric treatment
Molecular simulation of a supercapacitor pore	Integral or differential capacitance and ion occupancy	Charging time, voltage asymmetry, structural or spectroscopic data	Force-field error or an oversimplified electrode and electrolyte polarization model
Pore-network or homogenized electrode	Effective conductivity, relaxation time, and impedance	Comparison with image-based geometry and a resolved local model	Incorrect connectivity, tortuosity, or pore-level closure
Learning-based surrogate	Independent physical solver and hold-out experiment	Changed geometry, charge distribution, and operating range	Interpolation mistaken for physical generalization

## Data Availability

The original contributions presented in this study are included in the article. Further inquiries can be directed to the corresponding author.
